# A numerical investigation of the GRLW equation using lumped Galerkin approach with cubic B-spline

**DOI:** 10.1186/s40064-016-1773-9

**Published:** 2016-02-27

**Authors:** Halil Zeybek, S. Battal Gazi Karakoç

**Affiliations:** Department of Applied Mathematics, Faculty of Computer Science, Abdullah Gul University, 38080 Kayseri, Turkey; Department of Mathematics, Faculty of Science and Art, Nevsehir Haci Bektas Veli University, 50300 Nevsehir, Turkey

**Keywords:** GRLW equation, Lumped Galerkin method, Cubic B-spline, Solitary waves, Undular bore, 41A15, 65N30, 76B25

## Abstract

In this work, we construct the lumped Galerkin approach based on cubic B-splines to obtain the numerical solution of the generalized regularized long wave equation. Applying the von Neumann approximation, it is shown that the linearized algorithm is unconditionally stable. The presented method is implemented to three test problems including single solitary wave, interaction of two solitary waves and development of an undular bore. To prove the performance of the numerical scheme, the error norms $L_{2}$ and ${L_{\infty}}$ and the conservative quantities ${I_{1}}$, ${I_{2}}$ and ${I_{3}}$ are computed and the computational data are compared with the earlier works. In addition, the motion of solitary waves is described at different time levels.

## Background

The generalized regularized long wave (GRLW) equation, which discussed here, is based upon the regularized long wave (RLW) equation. The RLW equation was firstly derived from long waves propagating in the positive *x*-direction as a model for small-amplitude long waves on the surface of water in a channel by Peregrine (1966, 1967). Benjamin et al. (1972) introduced the RLW equation as a reasonable alternative model to the more common Korteweg-de Vries (KdV) equation. The KdV equation describes the long waves with assumption of small wave amplitude and large wave length in non-linear dispersive and many other physical systems. Later, the equal width (EW) wave equation was used by Morrison et al. (1984) as an alternative model to the RLW equation. So, the GRLW equation is related to the generalized equal width (GEW) wave equation and the generalized Korteweg-de Vries (GKdV) equation. These general equations are nonlinear wave equations with $(p+1)\hbox {th}$ nonlinearity and have solitary wave solutions, which are pulse-like.

The GKdV equation is given by

1 $$U_{t}+\varepsilon U^{p}U_{x}+\mu U_{xxx}=0,$$

the GEW equation is written as

2 $$U_{t}+\varepsilon U^{p}U_{x}-\mu U_{xxt}=0,$$

and the GRLW equation has the following form:

3 $$U_{t}+U_{x}+p(p+1) U^{p}U_{x}-\mu U_{xxt}=0,$$

in which physical boundary conditions $U\rightarrow 0$ as $x\rightarrow \pm \infty$, the subscripts *t* and *x* represent time and spatial differentiation, $\varepsilon$ and *p* is a positive integer, $\mu$ is positive constant. The boundary and initial conditions are taken

4 $$\begin{aligned} \begin{array}{lll} &{}U(a,t)=0,&{} \quad U(b,t)=0, \\ &{}U_{x}(a,t)=0,&{}\quad U_{x}(b,t)=0, \\ &{}U(x,0)=f(x), &{} \quad a\le x\le b, \end{array} \end{aligned}$$

where *f*(*x*) is a localized disturbance inside the interval [*a*, *b*] and it will be considered later. In the fluid problems, *U* implies the vertical displacement of the water surface or similar physical quantity. In the plasma applications, *U* is denoted as negative of the electrostatic potential. That’s why, the solitary wave solution of Eqs. (1), (2) and (3) helps us to understand the many physical phenomena with weak nonlinearity and dispersion waves such as nonlinear transverse waves in shallow water, ion-acoustic and magnetohydrodynamic waves in plasma and phonon packets in nonlinear crystals.

The RLW equation is obtained by taking $p=1$ in GRLW equation (3). Up to now, many numerical including finite elements and analytical solution techniques have been presented on the RLW equation. The RLW equation was investigated with the growth of an undular bore by Peregrine (1966). Morrison et al. (1984) proposed the approximate analytical technique for the scattering of solitary waves of the RLW equation. Galerkin approach with linear, quadratic and quintic B-spline was used by Doğan (2002), Gardner et al. (1995) and Dağ et al. (2006). Collocation method was set up by Raslan (2001) and Saka et al. (2011) with quadratic and both sextic and septic B-splines functions. Esen and Kutluay (2006) obtained the numerical solution of the RLW equation with lumped Galerkin method using quadratic B-spline. Galerkin method with extrapolation techniques has been implemented to the RLW equation by Mei and Chen (2012). Later on, the RLW equation has been solved numerically by using von Neumann technique based on parametric quintic splines (Lin 2014).

If $p=2$ in Eq. (3), the obtained equation is called as the modified regularized long wave (MRLW) equation. Finite element methods based on quintic, cubic and septic collocation were used for obtaining the numerical solution of the MRLW equation by Gardner et al. (1997), Khalifa et al. (2008) and Karakoç et al. (2014). Collocation method based on quintic B-spline functions with Rubin and Graves linearization technique was investigated for solving the MRLW equation by Karakoç et al. (2013). The MRLW equation was solved numerically by Ali (2009) using mesh free collocation method. Galerkin approach with cubic B-spline has been applied to MRLW equation by Karakoç et al. (2015).

When we consider the GRLW equation discussed here, there are some exact and numerical solution techniques on its. Hamdi et al. (2004) presented the exact solution technique. Numerical methods based on decomposition scheme, finite difference scheme and element free kp-Ritz were introduced for GRLW equation by Kaya (2004), EL-Danaf et al. (2014) and Guo et al. (2014). An approximate quasilinearization scheme was used to solve the GRLW equation with initial condition on the formation of undular bore by Ramos (2007). Roshan (2012) and Mohammadi (2015) have got the numerical results of the GRLW equation using finite element method based on Petrov Galerkin and exponential B-spline collocation. Also, Galerkin and lumped Galerkin method used here have been implemented to the EW, KdVB, Coupled KdV and MEW equations by Doğan (2005), Saka and Dağ (2009), Kutluay and Uçar (2013) and Esen (2006).

Inspired by the results of the applied numerical methods to similar type equations, we can say that lumped Galerkin approach is an accurate and efficient numerical technique. So, in this work, we have constructed the lumped Galerkin approach with cubic B-splines to get the numerical results of the GRLW equation.

## A lumped Galerkin method

Firstly, the solution domain limited to a finite interval [*a*, *b*] is divided into *N* equal subinterval by the points $$x_{m}$$ such that $$ a=x_{0}<x_{1}\ldots <x_{N}=b$$ and length $$h=\frac{b-a}{N}=(x_{m+1}-x_{m})$$. Prenter (1975) described the cubic B-spline functions $$\phi _{m}(x)$$, ( *m*= $$-1(1)$$$$N+1$$), at the nodes $$x_{m}$$ which form a basis over the interval [*a*, *b*] by

5 $$\begin{aligned} \begin{array}{l} \phi _{m}(x)=\frac{1}{h^{3}}\left\{ \begin{array}{ll} (x-x_{m-2})^{3}, \quad &{} x\in [x_{m-2},x_{m-1}], \\ {h^{3}}+3{h^{2}}(x-x_{m-1})+3h(x-x_{m-1})^{2}-3(x-x_{m-1})^{3}, \quad &{} x\in [x_{m-1},x_{m}], \\ {h^{3}}+3{h^{2}}(x_{m+1}-x)+3h(x_{m+1}-x)^{2}-3(x_{m+1}-x)^{3},\quad  &{} x\in [x_{m},x_{m+1}], \\ (x_{m+2}-x)^{3}, \quad &{} x\in [x_{m+1},x_{m+2}], \\ 0 &{} otherwise. \end{array} \right. \end{array} \end{aligned}$$

Each cubic B-spline $\phi _{m}$ covers four finite intervals, hence each finite interval $[x_{m},x_{m+1}]$ is covered by four splines. The approximate solution $U_{N}(x,t)$ is denoted in terms of the cubic B-splines by

6 $$\begin{aligned} U_{N}(x,t)=\sum _{j=-1}^{N+1}\phi _{j}(x)\delta _{j}(t),\ \end{aligned}$$

in which the unknown time-dependent quantities $\delta _{j}(t)$ will be computed by using the boundary and weighted residual conditions. Using the equality $h\eta =x-x_{m}$ such that $0\le \eta \le 1$, the finite interval $[x_{m},x_{m+1}]$ is converted to more easily workable interval [0, 1]. So, the cubic B-splines (5) depending on variable $\eta$ over the gap [0, 1] are reorganized with

7 $$\begin{aligned} \begin{array}{ll} \phi _{m-1}&=(1-\eta )^{3}, \\ \phi _{m}&=1+3(1-\eta )+3(1-\eta )^{2}-3(1-\eta )^{3}, \\ \phi _{m+1}&=1+3\eta +3\eta ^{2}-3\eta ^{3}, \\ \phi _{m+2}&=\eta ^{3}. \end{array} \end{aligned}$$

Here we should mention that except for $\phi _{m-1}(x),\phi _{m}(x),\phi _{m+1}(x)$ and $\phi _{m+2}(x)$, all cubic B-spline functions are null over the finite element [0, 1]. Thus, approximation function (6) in terms of element parameters $\delta _{m-1},\delta _{m},\delta _{m+1},\delta _{m+2}$ and B-spline element shape functions $\phi _{m-1},\phi _{m},\phi _{m+1},\phi _{m+2}$ can be expressed over the interval [0, 1] by

8 $$\begin{aligned} U_{N}(\eta ,t)=\sum _{j=m-1}^{m+2}\delta _{j}\phi _{j}. \end{aligned}$$

The nodal values of $U,U^{\prime },U^{\prime \prime }$ with respect to the time parameters $\delta _{m}$ are derived from B-splines (7) and trial function (8) as follows:

9 $$\begin{aligned} \begin{array}{ll} U_{m}=U(x_{m})=\delta _{m-1}+4\delta _{m}+\delta _{m+1}, \\ U_{m}^{\prime }=U^{\prime }(x_{m})=3(-\delta _{m-1}+\delta _{m+1}), \\ U_{m}^{\prime \prime }=U^{\prime \prime }(x_{m})=6(\delta _{m-1}-2\delta _{m}+\delta _{m+1}), \end{array} \end{aligned}$$

where the superscript $^{\prime }$ and $^{\prime \prime }$ symbolize first and second derivative to $\eta$, respectively. When applying the Galerkin’s approach with weight function *W*(*x*) to Eq. (3), the weak form of Eq. (3) is obtained as

10 $$\begin{aligned} \int _{a}^{b}W(U_{t}+U_{x}+p(p+1) U^{p}U_{x}-\mu U_{xxt})dx=0. \end{aligned}$$

Implementing the change of variable $x\rightarrow \eta$ to integral (10), which yields

11 $$\begin{aligned} \int _{0}^{1}W\left( U_{t}+\frac{1}{h} U_{\eta }+\frac{p(p+1) }{h}{\mathring{U}} ^{p}U_{\eta }-\frac{\mu }{h^{2}}U_{\eta \eta t}\right) d\eta =0, \end{aligned}$$

where ${\mathring{U}}$ is considered to be a constant over an element to simplify the integral. Applying partial integration once to (11), this leads to the following equality:

12 $$\begin{aligned} \int _{0}^{1}\left[ W(U_{t}+\frac{(1+\lambda )}{h} U_{\eta })+\beta W_{\eta }U_{\eta t}\right] d\eta =\beta WU_{\eta t}|_{0}^{1}, \end{aligned}$$

in which $\lambda =p(p+1) {\mathring{U}}^{p}$ and $\beta =\frac{\mu }{h^{2}}$. Substituting cubic B-splines (7) instead of the weight function *W*(*x*) and trial function (8) into integral equation (12) forms

13 $$\begin{aligned} \sum _{j=m-1}^{m+2}\left[ \left( \int _{0}^{1}\phi _{i}\phi _{j}+\beta \phi _{i}^{\prime }\phi _{j}^{\prime }\right) d\eta -\beta \phi _{i}\phi _{j}^{\prime }|_{0}^{1}\right] \dot{\delta }_{j}^{e}+\sum _{j=m-1}^{m+2}\left( \frac{(1+\lambda )}{h} \int _{0}^{1}\phi _{i}\phi _{j}^{\prime }d\eta \right) \delta _{j}^{e}=0, \end{aligned}$$

where $\delta ^{e}=(\delta _{m-1},\delta _{m},\delta _{m+1},\delta _{m+2})^{T}$ and the dot states differentiation to *t*, which can be written in matrix form by

14 $$\begin{aligned} \left[ A^{e}+\beta (B^{e}-C^{e})\right] \dot{\delta }^{e}+\frac{(1+\lambda )}{h} D^{e}\delta ^{e}=0. \end{aligned}$$

The element matrices are

$$\begin{aligned} A_{ij}^{e}&=  \int _{0}^{1}\phi _{i}\phi _{j}d\eta =\frac{1}{140}\left[ \begin{array}{cccc} 20 &{} 129 &{} 60 &{} 1 \\ 129 &{} 1188 &{} 933 &{} 60 \\ 60 &{} 933 &{} 1188 &{} 129 \\ 1 &{} 60 &{} 129 &{} 20 \end{array} \right] \\ B_{ij}^{e}&=  {} \int _{0}^{1}\phi _{i}^{\prime }\phi _{j}^{\prime }d\eta =\frac{1}{ 10}\left[ \begin{array}{cccc} 18 &{} 21 &{} -36 &{} -3 \\ 21 &{} 102 &{} -87 &{} -36 \\ -36 &{} -87 &{} 102 &{} 21 \\ -3 &{} -36 &{} 21 &{} 18 \end{array} \right] \\ C_{ij}^{e}&=  {} \phi _{i}\phi _{j}^{\prime }|_{0}^{1}=3\left[ \begin{array}{cccc} 1 &{} 0 &{} -1 &{} 0 \\ 4 &{} -1 &{} -4 &{} 1 \\ 1 &{} -4 &{} -1 &{} 4 \\ 0 &{} -1 &{} 0 &{} 1 \end{array} \right] \\ D_{ij}^{e}&=  {} \int _{0}^{1}\phi _{i}\phi _{j}^{\prime }d\eta =\frac{1}{20}\left[ \begin{array}{cccc} -10 &{} -9 &{} 18 &{} 1 \\ -71 &{} -150 &{} 183 &{} 38 \\ -38 &{} -183 &{} 150 &{} 71 \\ -1 &{} -18 &{} 9 &{} 10 \end{array} \right] \end{aligned}$$

with the subscript $i, j=m-1,m,m+1,m+2$. A lumped form of $\lambda$ calculated from $\left( \frac{U_{m}+U_{m+1}}{2}\right) ^{p}$ is

$$\begin{aligned} \lambda =\frac{p(p+1) }{2^{p}}\left( \delta _{m-1}+5\delta _{m}+5\delta _{m+1}+\delta _{m+2}\right) ^{p}. \end{aligned}$$

By considering together contributions from all elements, the matrix equation (14) takes the form

15 $$\begin{aligned} \left[ A+\beta (B-C)\right] \dot{\delta }+\frac{(1+\lambda )}{h} D\delta =0, \end{aligned}$$

where $\delta =(\delta _{-1},\delta _{0},...,\delta _{N},\delta _{N+1})^{T}$ is a nodal parameters. The *A*, *B*, *C* and $\lambda D$ are septa-diagonal matrices and their line of *m* is

$$\begin{aligned} \begin{array}{ll} A=\frac{1}{140}\left( 1,120,1191,2416,1191,120,1\right) ,\,\,B=\frac{1}{10} \left( -3,-72,-45,240,-45,-72,-3\right) , \\ \\ C=\left( 0,0,0,0,0,0,0\right) ,\,\, D=\frac{1}{20}\left( -1,-56,-245,0,245,56,1\right) , \\ \\ \lambda D=\frac{1}{20}\left( \begin{array}{l} -\lambda _{1},-18\lambda _{1}-38\lambda _{2},9\lambda _{1}-183\lambda _{2}-71\lambda _{3},10\lambda _{1}+150\lambda _{2}-150\lambda _{3}-10\lambda _{4}, \\ \qquad \qquad \qquad \quad71\lambda _{2}+183\lambda _{3}-9\lambda _{4},38\lambda _{3}+18\lambda _{4},\lambda _{4} \end{array} \right) \end{array} \end{aligned}$$

where

$$\begin{aligned} \begin{array}{l} \lambda _{1}=\frac{p(p+1) }{2^{p}}\left( \delta _{m-2}+5\delta _{m-1}+5\delta _{m}+\delta _{m+1}\right) ^{p}, \quad  \lambda _{2}=\frac{p(p+1) }{ 2^{p}}\left( \delta _{m-1}+5\delta _{m}+5\delta _{m+1}+\delta _{m+2}\right) ^{p}, \\ \lambda _{3}=\frac{p(p+1) }{2^{p}}\left( \delta _{m}+5\delta _{m+1}+5\delta _{m+2}+\delta _{m+3}\right) ^{p}, \quad  \lambda _{4}=\frac{p(p+1) }{2^{p}}\left( \delta _{m+1}+5\delta _{m+2}+5\delta _{m+3}+\delta _{m+4}\right) ^{p}. \end{array} \end{aligned}$$

Implementing the forward finite difference $\dot{\delta }=\frac{\delta ^{n+1}-\delta ^{n}}{\Delta{t}}$ and Crank–Nicolson approach $\delta =\frac{1}{ 2}(\delta ^{n}+\delta ^{n+1})$ to Eq.  (15), we obtain the matrix system

16 $$\begin{aligned} \left[ A+\beta (B-C)+\frac{(1+\lambda ) {\Delta{t}}}{2 h}D\right] \delta ^{n+1}=\left[ A+\beta (B-C)-\frac{(1+\lambda ) {\Delta{t}}}{2 h}D\right] \delta ^{n}. \end{aligned}$$

Using the boundary conditions given by Eq. (4), the $\left( N+3\right) \times \left( N+3\right)$ system (16) is reduced to $\left( N+1\right) \times \left( N+1\right)$ matrix system. Since the row m of *A*, *B*, *C* and *D* has seven elements, the system (16) comprises of the diagonal matrix with seven columns element (known as septa-diagonal matrix). The septa-diagonal matrix system can be solved by using Thomas algorithm (see subsection ). In this solution procedure, we need to two or three inner iterations $\delta ^{n*}=\delta ^{n}+\frac{1}{2}\left( \delta ^{n}-\delta ^{n-1}\right)$ at each time step to minimize the non-linearity. After all of these processes, we can easily achieve the recurrence relationship between two time steps *n* and $n+1$ which is an ordinary member of the matrix system (16)

17 $$ \gamma _{1}\delta _{m-3}^{n+1}+\gamma _{2}\delta _{m-2}^{n+1}+\gamma _{3}\delta _{m-1}^{n+1}+\gamma _{4}\delta _{m}^{n+1}+\gamma _{5}\delta _{m+1}^{n+1}+\gamma _{6}\delta _{m+2}^{n+1}+\gamma _{7}\delta _{m+3}^{n+1} =  \gamma _{7}\delta _{m-3}^{n}+\gamma _{6}\delta _{m-2}^{n}+\gamma _{5}\delta _{m-1}^{n}+\gamma _{4}\delta _{m}^{n}+\gamma _{3}\delta _{m+1}^{n}+\gamma _{2}\delta _{m+2}^{n}+\gamma _{1}\delta _{m+3}^{n},
$$

where

$$\begin{aligned} \begin{array}{ll} \gamma _{1}=\frac{1}{140}-\frac{3\beta }{10}-\frac{(1+\lambda ) \Delta t}{40 h }, &{}\gamma _{2}=\frac{120}{140}-\frac{72\beta }{10}- \frac{56(1+\lambda ) \Delta t}{40h}, \\ \gamma _{3}=\frac{1191}{140}-\frac{45\beta }{10}-\frac{245(1+\lambda ) \Delta t}{40h}, &{}\gamma _{4}=\frac{2416}{140}+\frac{240\beta }{10}, \\ \gamma _{5}=\frac{1191}{140}-\frac{45\beta }{10}+\frac{245(1+\lambda ) \Delta t}{40h}, &{}\gamma _{6}=\frac{120}{140}-\frac{72\beta }{10}+\frac{ 56(1+\lambda ) \Delta t}{40 h}, \\ \gamma _{7}=\frac{1}{140}-\frac{3\beta }{10}+\frac{(1+\lambda ) \Delta t}{40 h }. \end{array} \end{aligned}$$

To initiate the iteration, the initial vector $\delta ^{0}$ must be calculated by using the initial and boundary conditions. Also, using the relations at the knots ${U_{N}}(x_{m},0) = U(x_{m},0),\quad m = 0,1,\ldots ,N$ and derivative condition ${U_{N}^{\prime}}(x_{0},0) = {U^{\prime}}(x_{N},0) = 0$ together with a variant of the Thomas algorithm, the initial vector $\delta ^{0}$ can be easily computed from the following matrix form

$$\begin{aligned} \left[ \begin{array}{cccccccc} -3 &{} 0 &{} 3 &{} &{} &{} &{} &{} \\ 1 &{} 4 &{} 1 &{} &{} &{} &{} &{} \\ &{} &{} &{} \ddots &{} &{} &{} &{} \\ &{} &{} &{} &{} 1 &{} 4 &{} 1 &{} \\ &{} &{} &{} &{} -3 &{} 0 &{} 3 &{} \end{array} \right] \left[ \begin{array}{c} \delta _{-1}^{0} \\ \delta _{0}^{0} \\ \vdots \\ \delta _{N}^{0} \\ \delta _{N+1}^{0} \end{array} \right] =\left[ \begin{array}{c} U^{\prime }(x_{0},0) \\ U(x_{0},0) \\ \vdots \\ U(x_{N},0) \\ U^{\prime }(x_{N},0) \end{array} \right] . \end{aligned}$$

### The solution of septa-diagonal matrix system with Thomas algorithm

As used in Fortran program and given by Zaki (2000), the solution method of septa-diagonal matrix system with Thomas algorithm is expressed as follows: The septa-diagonal system can be written by

$$\begin{aligned} a_{i}\delta _{i-3}+b_{i}\delta _{i-2}+{c_{i}}\delta _{i-1}+d_{i}\delta _{i}+e_{i}\delta _{i+1}+f_{i}\delta _{i+2}+g_{i}\delta _{i+3}=h_{i},\quad i=0,1,\ldots ,N, \end{aligned}$$

and ${a_{0}} = {b_{0}} = {c_{0}} = {a_{1}} = {b_{1}} = {a_{2}}  = {g_{N-2}} = {g_{N-1}} = {f_{N-1}} = {g_{N}} = {f_{N}}  = {e_{N}} = 0.$ In the first step, the parameters are organized with

$$\begin{aligned}&\begin{array}{l} \alpha _{0}=b_{0},\quad \beta _{0}=c_{0},\quad \mu _{0}=d_{0},\quad \zeta _{0}=\frac{e_{0}}{\mu _{0}},\quad \lambda _{0}=\frac{f_{0}}{\mu _{0}},\quad \eta _{0}=\frac{g_{0}}{\mu _{0}},\quad \gamma _{0}=\frac{h_{0}}{\mu _{0}}, \end{array}\\&\begin{array}{l} \alpha _{1}=b_{1},\quad \beta _{1}=c_{1},\quad \mu _{1}=d_{1}-\beta _{1}\zeta _{0},\quad \zeta _{1}=\frac{e_{1}-\beta _{1}\lambda _{0}}{\mu _{1}},\quad \lambda _{1}=\frac{f_{1}-\beta _{1}\gamma _{0}}{\mu _{1}}, \\ \eta _{1}=\frac{g_{1}}{\mu _{1}},\quad \gamma _{1}=\frac{h_{1}-\beta _{1}\gamma _{0}}{\mu _{1}}, \end{array} \end{aligned}$$

and

$$\begin{aligned} \begin{array}{l} \alpha _{2}=b_{2},\quad \beta _{2}=c_{2}-\alpha _{2}\zeta _{0},\quad \mu _{2}=d_{2}-\lambda _{0}\alpha _{2}-\beta _{2}\zeta _{1}, \quad \zeta _{2}=\frac{e_{2}-\eta _{0}\alpha _{2}-\beta _{2}\lambda _{1}}{\mu _{2}}, \\ \lambda _{2}=\frac{f_{2}-\beta _{2}\eta _{1}}{\mu _{2}},\quad \eta _{2}=\frac{g_{2}}{\mu _{2}},\quad \gamma _{2}=\frac{h_{2}-\alpha _{2}\gamma _{0}-\beta _{2}\gamma _{1}}{\mu _{2}}. \end{array} \end{aligned}$$

As a second step, we calculate the following parameters

$$\begin{aligned} \alpha _{i}= b_{i} -a_{i}\zeta _{i-3},\quad \beta _{i}={c_{i}}-a_{i}\lambda _{i-3}-\alpha _{i}\zeta _{i-2},\quad \mu _{i}=d_{i}-a_{i}\eta _{i-3}-\lambda _{i-2}\alpha _{i}-\beta _{i}\zeta _{i-1},\\ \zeta _{i}= \frac{e_{i}-\eta _{i-2}\alpha _{i} -\beta _{i}\lambda _{i-1}}{\mu _{i}},\quad \lambda _{i}=\frac{f_{i}-\beta _{i}\eta _{i-1}}{\mu _{i}},\quad \eta _{i}=\frac{g_{i}}{\mu _{i}},\\ \gamma _{i}=  \frac{h_{i}-\beta _{i}\gamma _{i-1} -\alpha _{i}\gamma _{i-2}-a_{i}\gamma _{i-3}}{\mu _{i}},\quad {\text{for}} \;i=3,4,\ldots ,N. \end{aligned}$$

Now we obtain the solution

$$\begin{aligned} \delta _{i}= \gamma _{i}-\zeta _{i}\delta _{i+1} -\lambda _{i}\delta _{i+2}-\eta _{i}\delta _{i+3},\quad i=0,1,\ldots ,N-4,N-3,\\ \delta _{N-2}=  \gamma _{N-2}-\lambda _{N-2}\delta _{N} -\eta _{N-2}\delta _{N-1}, \quad \delta _{N-1}=\gamma _{N-1} -\eta _{N-1}\delta _{N},\quad \delta _{N}=\gamma _{N}. \end{aligned}$$

### Stability analysis

In order to determine the linear stability analysis of the numerical algorithm, we use the Fourier method and assume that the quantity $U^{p}$ in the non-linear term ${U^{p}}{U_{x}}$ of GRLW equation is locally constant. Substituting the Fourier mode $\delta _{m}^{n}=g^{n}e^{imkh}$ where *k* is mode number, *h* is the element size and $i = {\sqrt{-1}}$, into the scheme (17), which produces the following equality

18 $$\begin{aligned} \begin{array}{ll} \gamma _{1}g^{n+1}e^{i(m-3)kh}+\gamma _{2}g^{n+1}e^{i(m-2)kh}+\gamma _{3}g^{n+1}e^{i(m-1)kh}+\gamma _{4}g^{n+1}e^{imkh} \\ \qquad + \,\gamma _{5}g^{n+1}e^{i(m+1)kh} +\gamma _{6}g^{n+1}e^{i(m+2)kh}+\gamma _{7}g^{n+1}e^{i(m+3)kh}\\ \quad = \gamma _{7}g^{n}e^{i(m-3)kh}+\gamma _{6}g^{n}e^{i(m-2)kh}+\gamma _{5}g^{n}e^{i(m-1)kh} +\gamma _{4}g^{n}e^{imkh} \\ \quad \quad +\,\gamma _{3}g^{n}e^{i(m+1)kh}+\gamma _{2}g^{n}e^{i(m+2)kh}+\gamma _{1}g^{n}e^{i(m+3)kh}. \end{array} \end{aligned}$$

Now, if Euler’s formula [${e^{ikh}} = \cos \left( kh\right) +i\sin \left( kh\right)$] is used in Eq. (18) and this equation is simplified, we have the growth factor

19 $$\begin{aligned} g=\frac{a-ib}{a+ib}, \end{aligned}$$

where

20 $$\begin{aligned} \begin{array}{l} a=\left( \gamma _{7}+\gamma _{1}\right) \cos \left( 3kh\right) +\left( \gamma _{6}+\gamma _{2}\right) \cos \left( 2kh\right) +\left( \gamma _{5}+\gamma _{3}\right) \cos \left( kh\right) +\gamma _{4}, \\ b=\left( \gamma _{7}-\gamma _{1}\right) \sin \left( 3kh\right) +\left( \gamma _{6}-\gamma _{2}\right) \sin \left( 2kh\right) +\left( \gamma _{5}-\gamma _{3}\right) \sin \left( kh\right) . \end{array} \end{aligned}$$

The modulus of |*g*| is 1, so the linearized scheme is unconditionally stable.

## Numerical examples and results

In this section, we have applied the lumped Galerkin method to three test problems including single solitary wave, interaction of two solitary waves and development of an undular bore. These three examples are formed by using different values of initial condition. To demonstrate the efficiency and accuracy of the presented numerical scheme, the ${L_{2}}$ and ${L_{\infty}}$ error norms are calculated by using the solitary wave solution in Eq. (22) and the following equalities:

$$\begin{aligned} { L }_{2}=\left\| U^{exact}-U_{N}\right\| _{2}\simeq \sqrt{ h\sum \nolimits _{J=0}^{N}\left| U_{j}^{exact}-\left( U_{N}\right) _{j}\right| ^{2}},\\ \ { L }_{\infty }=\left\| U^{exact}-U_{N}\right\| _{\infty }\simeq \max _{j}\left| U_{j}^{exact}-\left( U_{N}\right) _{j}\right| . \end{aligned}$$

Furthermore, so as to indicate that the numerical approach keeps the properties related to mass, momentum and energy, we observe the changes of the invariants

21 $${I_{1}}=\int _{a}^{b}Udx,\qquad {I_{2}}=\int _{a}^{b}\left[ U^{2}+\mu (U_{x})^{2} \right] dx,\qquad {I_{3}}=\int _{a}^{b}\left[ U^{4}-\mu (U_{x})^{2}\right] dx. $$

The exact solution of GRLW equation given in Gardner et al. (1997) and Roshan (2012) has the form

22 $$U(x,t) = \root p \of {\frac{c(p+2)}{2p }\sec h^{2}\left[ \frac{p}{2} \sqrt{\frac{c }{\mu (c+1) }}\left( x-(c+1)t-x_{0}\right) \right] } $$

where $\root p \of {\frac{c(p+2)}{2p } }$ is amplitude, $c+1$ is the speed of the wave traveling in the positive direction of the *x*-axis, $x_{0}$ is arbitrary constant.

### The motion of single solitary wave

For this problem, we use the initial condition obtained by taking $$t=0$$ in Eq. (22). To coincide with papers Dağ et al. (2006), Gardner et al. (1997), Khalifa et al. (2008), Ali (2009), Karakoç et al. (2013), Roshan (2012) and Mohammadi (2015), the same values of $$\mu = 1$$, $${x_{0}}=40$$, $${x\in} \left[ 0,100 \right] $$ and different values of *p*, *c*, *h*, $${\Delta {t}}$$ are considered. The numerical computations are run from the time $$t=0$$ to time $$t=10$$ or $$ t=20$$.

Firstly, we choose the quantities $$p=2$$, $$c=1$$, $$h=0.2$$, $${\Delta {t}} = 0.025$$ and $$p=2$$, $$c=0.3$$, $$h=0.1$$, $${\Delta {t}} = 0.01$$. These values yield the $$ amplitude=1$$ and $$amplitude=0.54772$$. The obtained results are given in Tables 1 and 2. It is observed from Table 1 that the changes of the invariants are less than 0.04, 0.05 and 0.05 %, respectively. In Table 2, three invariants are nearly unchanged as the time processes. Moreover, The values of the error norms $$ {L_{2}}$$ and $${L_{\infty}}$$ are adequately small.

In the second case, we take the parameters $$p=3$$, $$c=1.2$$, $$h=0.1$$, $${\Delta{t}} = 0.025$$ and $$p=3$$, $$c=0.3$$, $$h=0.1$$, $${\Delta {t}} = 0.01$$. These produce the $$ amplitude=1$$ and $$amplitude=0.6$$. The calculated quantities are presented in Tables 3 and 4. As can be seen in Table 3, the changes of the invariants are less than 0.5, 0.7 and $$0.7\,\%$$ . Table 4 shows that three invariants are almost constant as the time increases. Also, we observe that the quantities of the error norms $$ {L_{2}}$$ and $${L_{\infty}}$$ are reasonably small, as expected.

**Table 1:** Invariants and errors for single solitary wave with $p = 2, c = 1, h = 0.2,{\Delta{t}} = 0.025,\mu  = 1,x\in \left[ 0,100\right]$

Time	${I_{1}}$	${{I_{2}}}$	${{I_{3}}}$	${L_{2}}\times 10^{3}$	${L_{\infty}}\times 10^{3}$
0	4.4428661	3.2998133	1.4142140	0.00000000	0.00000000
2	4.4429408	3.2999387	1.4143308	1.95082039	1.19160336
4	4.4430058	3.3000340	1.4144250	2.36484347	1.22370847
6	4.4430683	3.3001243	1.4145151	2.45181423	1.20000405
8	4.4431302	3.3002134	1.4146042	2.45030808	1.15204959
10	4.4431919	3.3003022	1.4146930	2.41750291	1.08099621

**Table 2:** Invariants and errors for single solitary wave with $p=2,c=0.3,h=0.1,\Delta t=0.01,\mu =1,x\in \left[ 0,100\right]$

Time	${I_{1}}$	${I_{2}}$	${I_{3}}$	${L_{2}}\times 10^{4}$	${L_{\infty}}\times10^{4}$
0	3.5820205	1.3450941	0.1537283	0.00000000	0.00000000
4	3.5820206	1.3450942	0.1537284	0.87664666	0.42835220
8	3.5820207	1.3450943	0.1537284	1.09331524	0.42259060
12	3.5820207	1.3450943	0.1537284	1.16711699	0.42542846
16	3.5820207	1.3450944	0.1537284	1.20368923	0.43881496
20	3.5820206	1.3450944	0.1537284	1.22736382	0.44722941

**Table 3:** Invariants and errors for single solitary wave with $p=3,c=1.2,h=0.1,{\Delta{t}}=0.025,\mu =1,x\in \left[ 0,100\right]$

Time	$I_{1}$	${{I_{2}}}$	${{I_{3}}}$	${L_{2}}\times 10^{3}$	${L_{\infty}}\times 10^{3}$
0	3.7971850	2.8812503	0.9729681	0.00000000	0.00000000
2	3.7980891	2.8826274	0.9747778	6.37523435	4.16206480
4	3.7989816	2.8839827	0.9760069	10.53160077	6.58017074
6	3.7998750	2.8853393	0.9771207	13.02367954	8.10106559
8	3.8007710	2.8867002	0.9782095	13.93740889	8.73017950
10	3.8016702	2.8880662	0.9792942	13.29108053	8.47810737

**Table 4:** Invariants and errors for single solitary wave with $p=3,c=0.3,h=0.1,{\Delta{t}}=0.01,\mu =1,x\in \left[ 0,100\right]$

Time	$I_{1}$	${I_{2}}$	${I_{3}}$	${L_{2}}\times 10^{4}$	${L_{\infty}}\times 10^{4}$
0	3.6776069	1.5657603	0.2268463	0.00000000	0.00000000
2	3.6776071	1.5657606	0.2268544	1.18720589	0.73102952
4	3.6776072	1.5657607	0.2268573	1.60659681	0.88913800
6	3.6776072	1.5657607	0.2268575	1.76861454	0.81537826
8	3.6776072	1.5657607	0.2268575	1.85663605	0.75460192
10	3.6776072	1.5657608	0.2268574	1.91332225	0.77992648

Thirdly, if $$p=4$$, $$c=4/3$$, $$h=0.1$$, $${\Delta{t}}=0.01$$ and $$p=4$$, $$c=0.3$$, $$ h=0.1$$, $${\Delta{t}}=0.01$$, the solitary wave has $$amplitude=1$$ and 0.6. The obtained results are reported in Tables 5 and 6. Table 5 denotes that the changes of the invariants are less than 0.2, 0.3 and $$0.3\,\%$$. On the other hand, this change is too little in Table 6. As in the parameters of $$p=2,3$$, the quantities of the error norms $${L_{2}}$$ and $${L_{\infty}}$$ are sensibly small.

**Table 5:** Invariants and errors for single solitary wave with $p=4,c=4/3,h=0.1,{\Delta{t}}=0.01,\mu =1,x\in \left[ 0,100\right]$

Time	${I_{1}}$	${I_{2}}$	${I_{3}}$	${L_{2}}\times 10^{3}$	${L_{\infty}}\times 10^{3}$
0	3.4687090	2.6716914	0.7292045	0.00000000	0.00000000
2	3.4690660	2.6722659	0.7305244	2.71272493	1.97322350
4	3.4694090	2.6728105	0.7309610	3.80159123	2.65902173
6	3.4697519	2.6733547	0.7313161	3.84205549	2.71392029
8	3.4700954	2.6738997	0.7316538	2.88903866	2.11361885
10	3.4704395	2.6744459	0.7319875	1.51139451	0.85758574

**Table 6:** Invariants and errors for single solitary wave with $p=4,c=0.3,h=0.1,{\Delta{t}}=0.01,\mu =1,x\in \left[ 0,100\right]$

Time	${I_{1}}$	${I_{2}}$	${I_{3}}$	${L_{2}}\times 10^{4}$	${L_{\infty}}\times 10^{4}$
0	3.7592865	1.7300236	0.2894191	0.00000000	0.00000000
2	3.7592871	1.7300246	0.2894498	1.91721709	1.20079691
4	3.7592873	1.7300248	0.2894559	2.45184081	1.44560973
6	3.7592874	1.7300249	0.2894566	2.70531310	1.21535724
8	3.7592874	1.7300250	0.2894569	2.90077790	1.31685490
10	3.7592875	1.7300251	0.2894570	3.08940237	1.44471990

**Fig. 1:**
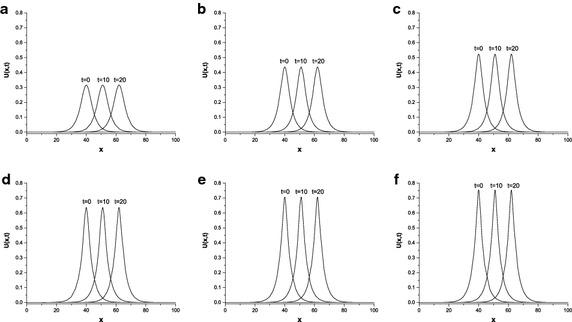
Single solitary wave with $c=0.1, {x_{0}}=40,x\in \left[ 0,100\right]$; **a** $p=2$, **b** $p=3$, **c** $p=4$, **d** $p=6$, **e** $p=8$, **f** $p=10$

Finally, we study the parameters $p=2,3,4,6,8,10$ with $c=0.03$ and $c=0.1$, $h=0.1$, ${\Delta{t}}=0.01$. The calculated values are listed in Table 7 which clearly shows that the error norms are sufficiently small and remain less than $5.2\times 10^{-3}$ with increasing time, *p* and *c*. In addition, the motion of single solitary wave is displayed at different times and the values of *p* in Fig. 1. From this figure, we can see that the solitary wave moves to the right at constant velocity and remains its shape and amplitude. When the values of *p* are increased, the peak position of single solitary wave rises.

**Table 7:** Errors for single solitary wave with $h=0.1,{\Delta{t}}=0.01,\mu =1,x\in \left[ 0,100\right]$

	p = 2		p = 3		p = 4		p = 6		p = 8		p = 10	
c	0.03	0.1	0.03	0.1	0.03	0.1	0.03	0.1	0.03	0.1	0.03	0.1
amp	0.17	0.31	0.29	0.43	0.38	0.52	0.52	0.63	0.60	0.70	0.66	0.75
Time												
$${L_{2}}\times 10^{4}$$												
5	4.36	0.16	5.84	0.37	6.89	0.65	8.26	1.44	9.12	2.76	9.71	5.09
10	5.15	0.27	6.91	0.52	8.15	0.88	9.78	2.24	10.80	5.61	11.53	13.26
15	5.28	0.36	7.08	0.63	8.35	1.08	10.02	3.25	11.08	9.92	11.91	27.67
20	5.54	0.44	7.43	0.74	8.77	1.29	10.53	4.51	11.67	15.92	12.66	51.36
$${L_{\infty}}\times 10^{4}$$												
5	2.21	0.09	2.96	0.21	3.49	0.36	4.18	0.82	4.61	1.68	4.90	3.20
10	2.11	0.13	2.83	0.25	3.33	0.43	4.00	1.18	4.41	3.09	4.68	7.34
15	2.01	0.16	2.69	0.29	3.18	0.51	3.81	1.66	4.20	5.12	4.46	14.39
20	4.16	0.19	5.57	0.34	6.58	0.61	7.88	2.22	8.69	7.88	9.23	25.82

In Table 8, we compare the quantity of invariants and error norms obtained by presented scheme with the ones given by earlier methods. From the table, we can conclude that three invariants are to be close to each other. The magnitude of our error norms is smaller than the ones given by Gardner et al. (1997), Khalifa et al. (2008), Ali (2009) and Roshan (2012) for $p=2$ and it is almost same with the paper (Roshan 2012) for $p=3,4$.

**Table 8:** Comprasions of result for the single solitary wave with $\mu =1,x\in \left[ 0,100\right]$

	Methods	$${L_{2}}\times 10^{3}$$	$${L_{\infty}}\times 10^{3}$$	$${I_{1}}$$	$$ {I_{2}}$$	$${I_{3}}$$
$p=2$	CBSC-CN (Gardner et al. 1995)	16.3900	9.2400	4.4420	3.2990	1.4130
$c=1$	CBSC+PA-CN (Gardner et al. 1995)	20.3000	11.2000	4.4400	3.2960	1.4110
$h=0.2$	CBSC (Khalifa et al. 2008)	9.3019	5.4371	4.4428	3.2998	1.4142
${\Delta{t}}=0.025$	MFC (Ali 2009)	3.9140	2.0190	4.4428	3.2997	1.4141
$t=10$	QBSPG (Roshan 2012)	3.0053	1.6874	4.4428	3.2998	1.4141
	QBSC (Karakoç et al. 2013)	2.4155	1.0797	4.4431	3.3003	1.4146
	EBSC (Mohammadi 2015)	2.3909	1.0647	4.4428	3.2998	1.4142
	Ours-CBSG	2.4175	1.0809	4.4431	3.3003	1.4146
	QBSPG (Roshan 2012)					
$p=3$	t = 1	0.0101	0.0080	3.6775	1.5657	0.2268
$c=0.3$	t = 5	0.0409	0.0238	3.6775	1.5657	0.2268
$h=0.1$	t = 10	0.0719	0.0377	3.6775	1.5657	0.2268
	Ours-CBSG					
${\Delta{t}}=0.01$	t = 1	0.0706	0.0514	3.6776	1.5657	0.2268
	t = 5	0.1702	0.0876	3.6776	1.5657	0.2268
	t = 10	0.1913	0.0779	3.6776	1.5657	0.2268
	QBSPG (Roshan 2012)					
$p=4$	t = 1	0.0158	0.0138	3.7592	1.7299	0.2894
$c=0.3$	t = 5	0.0542	0.0382	3.7592	1.7299	0.2894
$h=0.1$	t = 10	0.1225	0.0662	3.7592	1.7299	0.2894
	Ours-CBSG					
${\Delta{t}}=0.01$	t = 1	0.1222	0.0983	3.7592	1.7300	0.2894
	t = 5	0.2591	0.1357	3.7592	1.7300	0.2894
	t = 10	0.3089	0.1444	3.7592	1.7300	0.2894

### The interaction of two solitary waves

In the second test problem, we have worked on

23 $$\begin{aligned} U(x,0)=\sum _{i=1}^{2}\root p \of {\frac{{c_{i}}(p+2)}{2p }\sec h^{2} \left[ \frac{p}{2} \sqrt{\frac{{c_{i}}}{\mu ({c_{i}}+1)} }(x-x_{i})\right] }, \end{aligned}$$

which provides two positive solitary waves having different amplitudes of magnitudes 2 and 1 at the same direction, where $${c_{i}}$$ and $${x_{i}}$$, $$ i=1,2$$ are arbitrary constants.

**Fig. 2:**
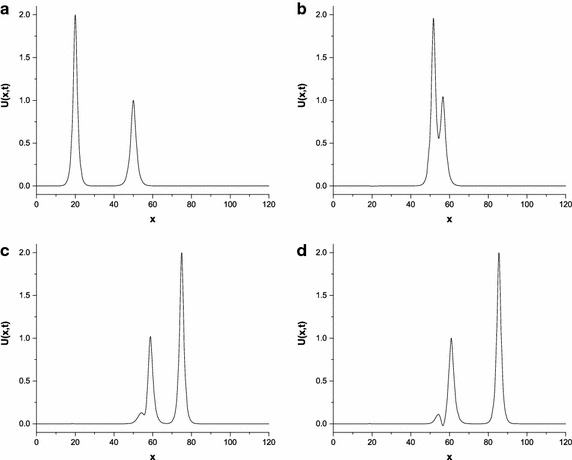
Interaction of two solitary waves at $$p=3$$; **a** $$t=0$$, **b** $$t=3$$, **c** $$ t=5$$, **d** $$p=6$$

**Fig. 3:**
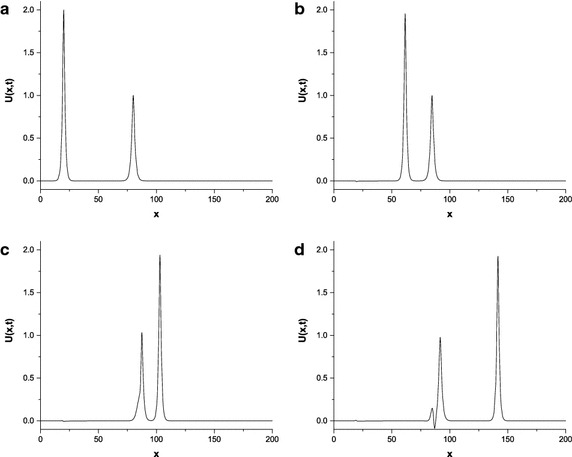
Interaction of two solitary waves at $$p=4$$; **a** $$t=0$$, **b** $$t=2$$, **c** $$ t=4$$, **d** $$t=6$$

The parameters are chosen to be first values $p=2$, $c_{1}=4$, $c_{2}=1$, $x_{1}=25$, $x_{2}=55$, $h=0.2$, ${\Delta{t}}=0.025$, $\mu =1$, $x\in \left[ 0,250\right]$; second values $p=3$, $c_{1}=48/5$, $c_{2}=6/5$, $x_{1}=20$, $x_{2}=50$, $h=0.1$, ${\Delta{t}}=0.01$, $\mu =1$, $x\in \left[ 0,120\right]$ and third values $p=4$, $c_{1}=64/3$, $c_{2}=4/3$, $x_{1}=20$, $x_{2}=80$, $h=0.125$, ${\Delta{t}}=0.01$, $\mu =1$, $x\in \left[ 0,200\right]$. The numerical computations are given in Tables 9 and 10. The results in Tables show that the changes of the invariants from their initial state are as small as required and good agreement with those of Roshan (2012). The motion of two solitary waves is simulated at different time levels in Figs. 2 and 3. These figures show that the initial position of the wave with larger amplitude is on the left of the second wave with smaller amplitude. As the time processes, the large wave catches up with the smaller one and overlapping process occurs. After a while, waves start to resume their original forms.

**Table 9:** Invariants for interaction of two solitary waves with $p=2,c_{1}=4,c_{2}=1,x_{1}=25,x_{2}=55,h=0.2,{\Delta{t}}=0.025,\mu =1,x\in \left[ 0,250\right]$

Time	${I_{1}}$		${I_{2}}$		${I_{3}}$	
	Ours-CBSG	QBSPG (Roshan 2012)	Ours-CBSG	QBSPG (Roshan 2012)	Ours-CBSG	QBSPG (Roshan 2012)
0	11.4676	11.4677	14.6290	14.6286	22.8804	22.8788
4	11.4674	11.4677	14.6287	14.6292	22.8783	22.8811
8	11.4685	11.4677	14.6360	14.6229	22.9020	22.8798
12	11.4663	11.4677	14.6257	14.6299	22.8717	22.8803
16	11.4664	11.4677	14.6260	14.6295	22.8686	22.8805
20	11.4662	11.4677	14.6253	14.6299	22.8650	22.8806

**Table 10:** Invariants for interaction of two solitary waves with $p = 3$ and 4

Time	0	1	2	3	4	5	6
p=3							
${I_{1}}$	9.6907	9.6907	9.6906	9.6917	9.6898	9.6898	9.6901
${I_{2}}$	12.9443	12.9443	12.9440	12.9489	12.9418	12.9420	12.9426
${I_{3}}$	17.0187	17.0311	17.0324	18.0050	16.9849	16.9222	16.9557
p = 4							
${I_{1}}$	8.8342	8.7559	8.7089	8.6774	8.6518	8.6322	8.6134
${I_{2}}$	12.1707	11.9304	11.7871	11.6932	11.6179	11.5560	11.4992
${I_{3}}$	14.0296	13.3472	12.9204	13.2047	12.1972	12.0924	11.9640

### The development of an undular bore

**Fig. 4:**
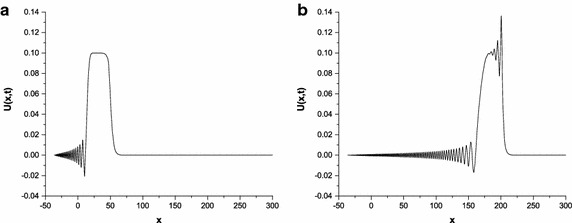
Solution profiles of the undular bore at $p=2$; **a** $t=50$, **b** $t=200$

**Fig. 5:**
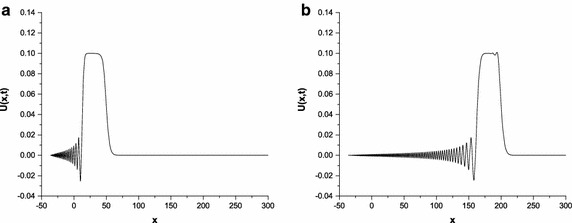
Solution profiles of the undular bore at $p=3$; **a** $t=50$, **b** $t=200$

**Fig. 6:**
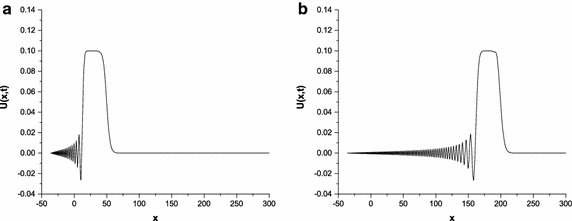
Solution profiles of the undular bore at $p=4$; **a** $t=50$, **b** $t=200$

As a last test problem, we have focused on the development of an undular bore given by

24 $$\begin{aligned} U(x,0)=\frac{1}{2 } {U_{0}}\left[ 1-\tanh \left( \frac{x-x_{c}}{d}\right) \right] , \end{aligned}$$

which indicates the elevation of the water above the equilibrium surface at time zero. The change in water level of magnitude Eq. (24) is centered on $x=x_{c}$. We study with the parameters ${U_{0}}=0.1, \mu =1/6, x_{c}=0, d=5, h=0.1, {\Delta{t}}=0.1, x\in \left[ -36,300\right]$ to be consistent with earlier works (Peregrine 1966; Esen and Kutluay 2006; Mei and Chen 2012; Doğan 2005). The conservative quantities are recorded in Table 11. In this table, the changes of the invariants remain less than $1.1\times 10^{-2}$, $1.0\times 10^{-3}$ and $2.0\times 10^{-3}$, respectively. The undulation profiles are depicted at time $t=50$ and $t=200$ when $p=2,3,4$ in Figs. 4, 5 and 6. It is understood that the magnitude of the waves increases with rising the value of *x*. Later, undulations take the peak position and disappear.

**Table 11:** Invariants for development of an undular bore

Time	${I_{1}}$			${I_{2}}$			${I_{3}}$		
	p = 2	p = 3	p = 4	p = 2	p = 3	p = 4	p = 2	p = 3	p = 4
Our results for ${U_{0}}=0.1,x_{0}=0,d=5,\mu =1/6,h=0.1,{\Delta{t}}=0.1,x\in \left[ -36,300\right]$									
0	3.5949	3.5949	3.5949	0.3344	0.3344	0.3344	0.0031	0.0031	0.0031
50	3.6051	3.6050	3.6049	0.3348	0.3350	0.3350	0.0019	0.0016	0.0015
100	3.6051	3.6050	3.6050	0.3348	0.3350	0.3350	0.0018	0.0016	0.0015
150	3.6050	3.6050	3.6049	0.3350	0.3349	0.3350	0.0017	0.0016	0.0015
200	3.6050	3.6050	3.6049	0.3354	0.3349	0.3350	0.0012	0.0016	0.0015

Time	${I_{1}}$			${I_{2}}$			${I_{3}}$		
	p = 2			p = 2			p = 2		
QBSC[28] results for ${U_{0}}=0.1,d=5,\mu =3/2,h=0.2,{\Delta{t}}=0.1,x\in \left[ 0,250\right]$									
0	4.0000			0.3759			0.0025		
50	4.8507			0.4620			0.0034		
100	5.7016			0.5480			0.0042		
150	6.5531			0.6341			0.0051		
200	7.4055			0.7204			0.0060		

## Conclusion

The solitary-wave solutions of the GRLW equation have been successfully obtained by using lumped Galerkin method based on cubic B-spline functions. Also, the linearized scheme has been found to be unconditonally stable. The error norms ${L_{2}}$, ${L_{\infty}}$ and three conservative quantities ${I_{1}}$, ${I_{2}}$ and ${I_{3}}$ have been computed for single solitary wave, interaction of two solitary waves and development of an undular bore. These computations demonstrate that our error norms are as small as required and they are smaller than the most of existing numerical calculations or too close to the best result in literature. The numerical algorithm conserves the properties related to mass, momentum and energy and the numerical values of them have been found to be in good agreement with earlier studies. In addition, the profiles of the solitary wave are similar to those of references. As a result, we can say that lumped Galerkin method is more practical, accurate and productive numerical approximation technique for GRLW equation and it can be reliably used to solve the similar type non-linear problems.
